# The recent state of a hundred years old classic hypothesis of the cerebrospinal fluid physiology

**DOI:** 10.3325/cmj.2017.58.381

**Published:** 2017-12

**Authors:** Darko Orešković, Milan Radoš, Marijan Klarica

**Affiliations:** 1 Ruđer Bošković Institute, Department of Molecular Biology, Zagreb, Croatia *doresk@irb.hr*; 2 Department of Pharmacology and Croatian Institute for Brain Research, School of Medicine University of Zagreb, Zagreb, Croatia

The knowledge of cerebrospinal fluid (CSF) physiology is mainly presented in hypotheses. Today there are two relevant hypotheses on CSF physiology; the classic one, known as the third circulation (1,2), and the new one (3-5). The classic hypothesis is still predominantly present in medical and scientific literature, and new experimental-based knowledge is unfortunately still not sufficiently involved in discussions about CSF physiology. However, we hope that in time, the framework of thinking associated with education within the classic hypothesis is becoming more and more tolerable and that the hypothesis, which corresponds to experimental and clinical results, and physiological relationships in the central nervous system will take an objective place in CSF physiology.

Seven years ago, after more than 30 years of our experimental work in the field of CSF physiology, we were the first research group that completely abandoned the classic CSF hypothesis and proposed a new one explaining the relationships between CSF, interstitial fluid (ISF), surrounding tissue of CSF system and blood, based on experimental data, clinic evidence, and relevant scientific publications (3-5). According to our hypothesis, CSF secretion, circulation, and absorption do not exist. Instead, CSF appears and disappears (exchanges) everywhere within the CSF system, ie, inside the cranium and spinal part (3-5). The main regulators of CSF and ISF volumes, which are connected and can be observed as a functional unit, are osmotic and hydrostatic forces related to the vast capillary network of the brain and the spinal cord (3-5). Despite such significant changes in the understanding of CSF physiology, most authors still attempt to present their results in line with the classic hypothesis.

## The example of misinterpretation of results in accordance with classic hypothesis of CSF physiology

The regulation of intracranial pressure (ICP) was explained by the classic hypothesis of CSF physiology. It is believed that ICP is regulated by the relationship between CSF secretion, circulation, and absorption. During permanent active CSF formation (secretion), ICP depends on the resistance to CSF flow (circulation) throughout the CSF system and resistance to CSF absorption into the blood. This means that a higher resistance should result in higher ICP. ICP alterations during the changes of body position could also be interpreted in the same way. Furthermore, ICP varies with body position (6-11), but little is known about the mechanisms that are controlling these variations. Thus, in the new study by Holmlund et al (12) and other similar research, the correlation between ICP during verticalization and classic hypothesis was presented. The authors reported their research on internal jugular veins (IJVs) by ultrasound imagining in the supine and sitting position on healthy volunteers, and observed the partial collapse of IJVs in an upright posture, which they tried to explain and describe as a mechanism for postural intracranial pressure (ICP) regulation. The relationship between the classic hypothesis and hypothesis of ICP regulation proposed by Holmlund et al (12) was presented as follows:

ICP = I_form_ x R_out +_ P_dural_ Equation 1

where I_form_ = the rate of CSF secretion; R_out_ = the CSF outflow resistance, and P_dural_ = the pressure in the dural veins. The Equation 1 for ICP calculation is based on the classic hypothesis because it states that the ICP mainly depends on CSF secretion and absorption by the dural sinuses. Furthermore, Equation 1 shows that the change of body position cannot directly influence the ICP, which means that the observed change of ICP after verticalization can only result from the changes in CSF secretion or absorption rates and/or dural pressure (P_dural_) changes. Hence, if someone would like to study the influence of body posture on ICP, they should determine which of the mentioned parameters are affected. In Holmlund et al’s article (12),a partial collapse of IJVs in an upright position was proposed to regulate the pressure inside the dural veins (P_dural_), and, consequently, ICP. Finally, the authors explained the experimental results and the mechanism of ICP regulation during changes of body position exclusively in accordance with the classic CSF hypothesis.

We believe that the ICP regulation cannot be explained using the proposed Equation 1 in which those parameters are crucial for ICP calculation. Our new approach to CSF physiology has also required re-evaluation of traditional hypothesis of ICP regulation.

Re-evaluation of the traditional hypothesis of ICP and proposition of the new one resulted from an animal (cat) and phantom experiment (“plastic-rubber” model) (9-11). It was clearly shown on cats that ICP in horizontal position is the same in the cranial and spinal subarachnoid space (about +14.0 cm H_2_O), but after verticalization becomes sub-atmospheric (about -4.0 cm H_2_O) inside the cranium and highly positive (about +30.0 cm H_2_O) in thw lumbar subarachnoid space (9-11). This difference (between -4.0 cm H_2_O and +30.0 cm H_2_O) in the vertical position produces a constant hydrostatic pressure gradient, also observed in humans (6-8). The study of verticalization effects on ICP in the phantom experiment (“plastic-rubber” model, which by its anatomical dimensions and basic biophysical features imitates the craniospinal system in cats) has faithfully imitated the results obtained in cats. Similar values were recorded in both cranial and spinal subarachnoid spaces (about +12.0 cm H_2_O) in horizontal position, while negative ICP values were measured inside the cranium (about -4.0 cm H_2_O) and highly positive ones (about +30.0 cm H_2_O) inside the lumbar space in an upright position (11). Thus, after verticalization, the values of hydrostatic pressure differ enormously (from -4.0 cm H_2_O to +30.0 cm H_2_O) both in the phantom experiment and in cats. These values increase in linear fashion with each distance unit from the measuring point inside the cranium toward the lumbar region (11). Hence, the observed hydrostatic ICP gradient could not be explained by Equation 1, which allows for calculation of only one unique ICP inside the CSF system, which in vertical position does not correspond to the real situation. Moreover, ICP in patients is generally measured inside the lumbar CSF space in a supine/recumbent (horizontal) position, and that hydrostatic pressure value (which is the same in the lumbar and cranial parts) is called ICP, although obtained value is not measured inside the cranium. Therefore, a term “CSF pressure (CSFP)” instead of ICP would be better used in situations in which the hydrostatic pressure is measured in the CSF system, because the change of body position does not relate only to hydrostatic pressure inside the cranium, but to the whole CSF system.

We did not observe the difference in behavior of CSFP between experimental animals and phantom after verticalization. In phantom, there were no biological and physiological influences, and “CSFP” was defined only by the laws of fluid mechanics. We concluded that CSFP in experimental animals also depended on the laws of fluid mechanics and anatomical characteristics of the cranial and spinal CSF spaces (11) rather than on CSF secretion, circulation, and absorption, as still generally accepted.

The presented hypothesis of the CSFP regulation takes into account the CSF pressure inside the entire CSF system (cranial and spinal spaces) as a unique department in which the changes of pressure in one part of the system are inextricably linked to the changes in the other (11). The hypothesis has been theoretically confirmed by the fluid mechanics equation and complemented by the literature data (11).

Our research (9-11) is very similar to the research described by Holmlund et al (12), although in our experiments, we did not measure the collapse of IJVs. However, if the IJVs collapse represents a mechanism by which CSFP is regulated after verticalization (12), than it should also be present in cat experiments during CSFP regulation after verticalization (9-11). Respectively, if the collapse of the IJVs really regulates the CSFP, then after the verticalization equal CSFP change in cats should not be obtained as in phantom, where no veins (IJVs) exist at all (11). Still, our results were obtained on animals (9-11) and results of Holmlund et al (12) on humans, and one should always be very careful in translating the results from animal species to humans. However, the fact is that the knowledge on human CSF physiology is mostly based on animal experiments and, in discussed study (12), the authors use Equation 1 derived from the experiments performed on rabbits to explain the observed collapse of IJV as a regulator of CSFP in humans.

## Conclusion

According to our results (9-11), the key effect on CSFP during changes of body position in physiological conditions is the effect of gravitation force on the CSF column, which is disposed inside the whole (cranial and spinal part) CSF system. This means that the regulation of CSFP during verticalization is caused by both the law of fluid mechanics and biophysical characteristics of cranial and spinal intradural space. The changes in venous system, the size of imagined CSF secretion, the resistance to hypothetical CSF circulation and CSF absorption, and the hydrostatic pressure inside the dural sinuses do not have any visible impact on CSFP in physiological conditions. Since the verticalization in cats and in phantom produces the same effect on CSFP, and considering the difference between the CSFP values in cats where the IJVs were affected and in phantom where IJVs do not exist, both clearly suggest that IJVs are not involved in CSFP regulation. In other words, in physiological conditions, acute changes in venous pressure during verticalization seem to be independent of the acute change of CSFP and that conclusion about the interaction between CSFP and partial collapse of IJVs should be more carefully formulated. In our opinion, the collapse of IJVs is not the cause of CSFP regulation, but rather a consequence of the laws of fluid mechanics, which should be the subject of additional studies and analysis.

Finally, we should not lose sight of what the father of experimental medicine, Claude Bernard said: “*Hypotheses are only theories, verified by more or less numerous facts. Those verified by the most facts are the best, but even then they are never final, never to be absolutely believed.”* In other words, the hypotheses, including the classic one, should last so long as experimental and clinical data fit into their framework.
